# Acceptability, motivation and the prospect of cure for people living with HIV and their healthcare providers in HIV cure-focused treatment interruption studies

**DOI:** 10.1186/s12981-020-00321-z

**Published:** 2020-11-10

**Authors:** Jillian S. Y. Lau, Miranda Z. Smith, Brent Allan, Cipriano Martinez, Jennifer Power, Sharon R. Lewin, James H. McMahon

**Affiliations:** 1 Department of Infectious Diseases, Alfred Health and Monash University, Melbourne, Australia; 2 The Peter Doherty Institute for Infection and Immunity, University of Melbourne and Royal Melbourne Hospital, Melbourne, Australia; 3 International Council of AIDS Service Organizations, Toronto, Canada; 4 Australasian Society for HIV, Viral Hepatitis and Sexual Health Medicine, Sydney, Australia; 5 National Association of People Living with HIV Australia, Sydney, Australia; 6 Australian Research Centre for Sex Health and Society, La Trobe University, Melbourne, Australia

**Keywords:** HIV cure, Analytical treatment interruption, Clinical studies, Social survey, Acceptability

## Abstract

**Background:**

Analytical treatment interruptions (ATI) are commonly used clinical endpoints to assess interventions aimed at curing HIV or achieving antiretroviral therapy (ART)-free HIV remission. Understanding the acceptability of ATI amongst people living with HIV (PLHIV) and their HIV healthcare providers (HHP) is limited.

**Methods:**

Two online surveys for PLHIV and HHP assessed awareness and acceptability of ATI, and understanding of the prospect for HIV cure in the future. Responses were collected from July 2017–January 2018. A descriptive analysis was performed and similar questions across the two surveys were compared using χ squared test.

**Results:**

442 PLHIV and 144 HHP completed the survey. 105/400 (26%) PLHIV had ever interrupted ART, 8% of which were in a clinical trial. Altruistic motivations were drivers of participation of PLHIV in cure related research. 81/135 (60%) HHP would support their patients wishing to enrol in an HIV cure-focused trial, but fewer would promote and allow such participation (25% and 31% respectively). Compared to HHP, PLHIV were more likely to believe that an HIV cure would be achievable within 10 years (55% vs. 19%, p < 0.001), had less awareness of ATI (46% vs. 62%, p < 0.001) and were less likely to have had experience of either participation or enrolment in an ATI study (5% vs. 18%, p < 0.001)

**Conclusion:**

PLHIV were more optimistic about the potential for HIV cure. HHP had more direct experience with HIV cure-focused studies. Educational strategies are required for both groups to increase understanding around ATIs in HIV cure research but should be tailored specifically to each group.

## Introduction

Analytical treatment interruptions (ATI) are structured and temporary cessations of antiretroviral therapy (ART), performed in the context of HIV cure-focused clinical studies. ATI is increasingly being used to assess the effects of interventions aimed at achieving durable virological control off antiretroviral therapy (ART) [1]. This rising number of studies utilising ATI has led to much debate about how ATI should be performed. As such, recent consensus guidelines written by key stakeholders have outlined principles for ATI design to produce robust and ethically sound scientific outcomes with minimal risk to the participant [2].

Despite great interest in ATI, understanding of ATI acceptability amongst people living with HIV (PLHIV) and their HIV healthcare providers (HHP) is limited. In previous surveys of PLHIV, acceptability of ATI has ranged from 34 to 68% [3–5], and small qualitative studies have explored understanding and acceptance of ATI [6, 7]. Knowledge of healthcare provider acceptance of, or support for HIV cure-focused research is even more limited. While Protiere et al. have explored clinician attitudes towards HIV cure research in France [7]; studies specifically into clinician perceptions towards ATI have not been performed. Media reporting of HIV cure science has also lacked commentary from PLHIV [8].

We have previously published a study reviewing PLHIV and HHP acceptability of ATI methodology (including monitoring frequencies and thresholds to restart therapy) and perceived risks of ATI in HIV cure-focused research [9]. Here we describe the acceptability and previous experience with ATI in both PLHIV and HHP, and PLHIV motivations to enrol in cure-focused clinical trials where ART is interrupted.

## Methods

In collaboration with the Australian HIV Cure Community Partnership, and peak HIV research and advocacy organisations (National Association of People Living with HIV Australia, The Peter Doherty Institute for Infection and Immunity, The Alfred Hospital, Positive Living Victoria, and the Australian Research Centre for Sex, Health and Society), two online surveys (appendix 1 and 2) were formulated and hosted online at HIVcure.com.au [10]. Links to the surveys were disseminated through mailing lists, newsletters and social media of various professional organisations and HIV community advocacy groups. These organisations have been listed as acknowledged parties. The survey for PLHIV (38 questions) assessed previous participation in HIV research and factors affecting motivation to enrol in HIV cure-focused studies, and any past experience with ART interruption. The HHP survey (16 questions) evaluated interest in HIV cure-focused research, previous experience enrolling patients into HIV cure-related studies, and support for participation in such studies. Both surveys explored awareness of ATI, and anticipated timeframes for a cure for HIV being achieved. Participant information was collected on self-reported demographics. Further details on the development of the surveys have been described previously [9].

The survey platform allowed responses to be entered only once from a specific computer or device, which minimised the risk of invalid or spam responses. Participants were not offered any financial incentive to complete the survey and were not reimbursed in any way for their time. Ethics approval was granted by the Alfred Hospital Human Research Ethics Committee (approval no. 312/17), and all participants checked an electronic consent prior to proceeding to the survey questions. Responses were collected from July 2017–January 2018.

Responses were recorded on a four-point Likert scale from “not at all important” to “very important” for factors affecting PLHIV motivation to enrol in HIV cure-related studies, and HHP support for enrolment in HIV cure-related studies (“definitely” to “would not support”). The samples of PLHIV and HHP were not linked. Responses to questions that were asked of both PLHIV and HHP were compared by chi squared test, with significance calculated at a p value of < 0.05.

## Results

The community survey was completed by 442 PLHIV: 273 (78%) respondents were male, 222 (64%) identified as gay, lesbian or homosexual. 80% resided in urban or metropolitan areas in high- or middle-income countries in North America, Western Europe and Australasia. 144 HHP completed the provider survey, of which 101 (72%) practiced in Australia. 72 (51%) worked in tertiary teaching hospitals, and 33 (23%) practised in primary care clinics. Further demographic details on participants have been reported previously [9].

### PLHIV

**Fig. 1:**
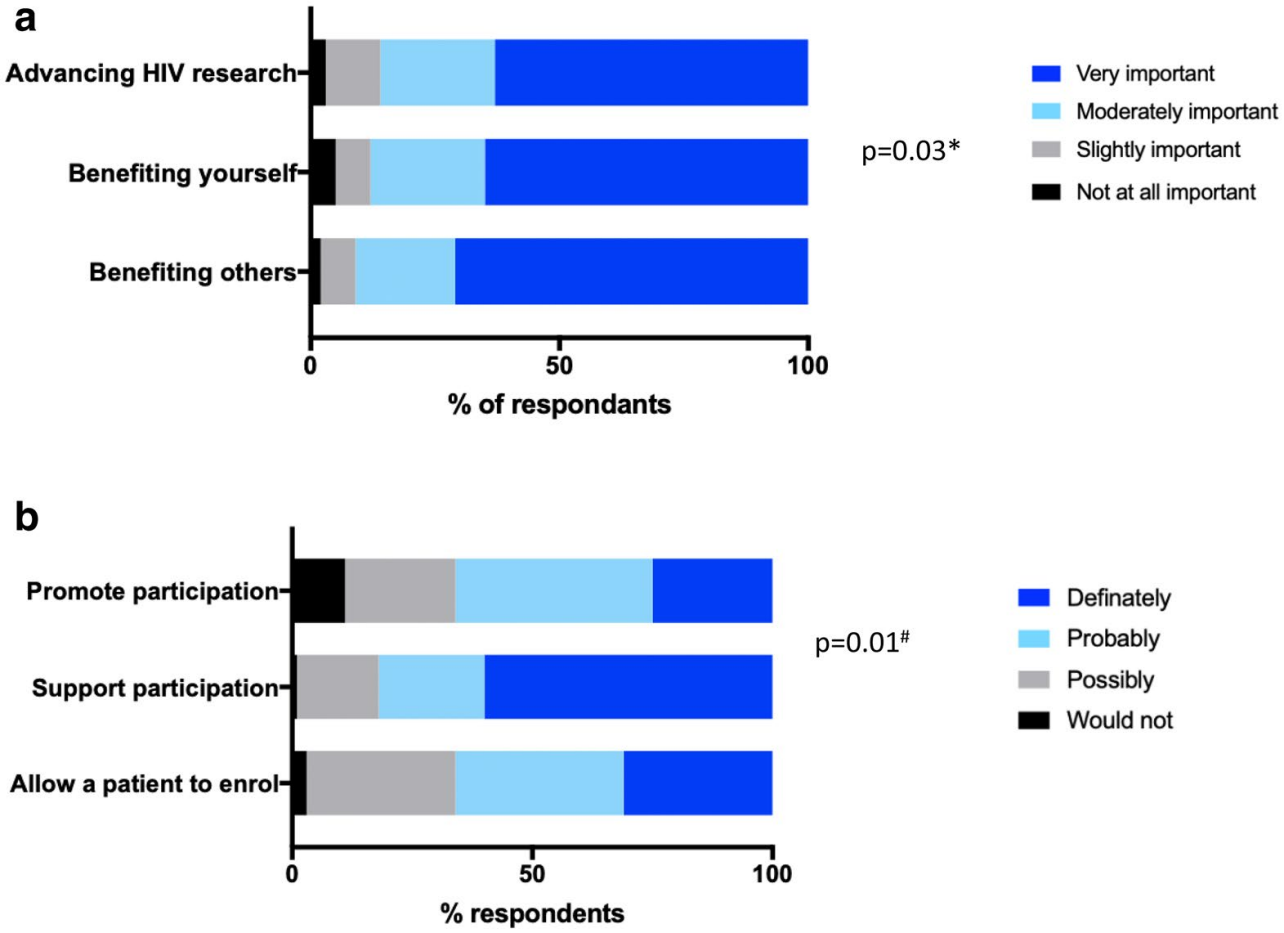
Factors affecting enrolment in HIV cure-focused trials in **a** people living with HIV and **b** HIV healthcare providers. *Comparing “Very important” to the other responses across the 3 questions. ^#^Comparing “Definitely” to the other responses across the 3 questions

194/419 (46%) PLHIV had previously participated in any kind of HIV research, including social surveys, and 150/410 (37%) had enrolled in a HIV clinical trial. Only 21/412 (5%) had previously participated in an HIV cure-focused study though 182/399 (46%) were aware of the practice of ATI in HIV cure-focused clinical trials. 105/400 (26%) had ever interrupted ART; reasons included own choice (36%), on clinician advice (5%), cost (8%), for a study (8%), side effects (21%) or other (22%). When asked what motivated them to enrol in HIV cure-focused clinical trials, “benefiting others”, “advancing HIV research”, and “benefiting myself” were selected as a “very important” motivation (highest on the four-point likert scale) by 285/399 (71%), 251/398 (63%) and 260/399 (65%) of respondents respectively (p = 0.03 for very important compared to other responses across all 3 questions, Fig. 1a). Only 37/355 (10%) PLHIV stated that they would *never* interrupt ART for a study.

### HHP

85/140 (61%) respondents to the HHP survey stated that they were “very interested” in HIV cure-focused studies. Only 4/140 (3%) had *never* heard of research directed towards an HIV cure. 25/140 (18%) HHP had previously enrolled a patient of theirs into an HIV cure-focused clinical trial. 81/135 (60%) would “definitely” support their patients if they wished to learn more about clinical trials involving ATI, or wished to enrol in such a trial. However only 42/137 (31%) stated they would “definitely” allow their patient to enrol in a study with ATI, and only 34/137 (25%) would “definitely” promote such studies to their patients (p < 0.001 for definitely compared with other responses across all 3 questions, Fig. 1b).

### Compared responses from PLHIV and HHP

Responses from questions comparable between PLHIV and HHP are summarised in Table 1. Higher optimism for cure was demonstrated amongst PLHIV, with 226/410 (55%) PLHIV thinking a cure was achievable within 10 years compared to 26/140 (19%) HHP (p < 0.001). 86/138 (62%) HHP were aware of the role of ATI to test HIV cure-focused interventions, compared with 182/399 (46%) PLHIV (p < 0.001). More HHP, 25/140 (18%), had enrolled someone into an HIV cure-focused study relative to 21/412 (5%) PLHIV who had ever participated in such a study (p < 0.001).

**Table 1:** Community and provider attitudes towards analytical treatment interruptions

	Comparable responses between community and providers				
	PLHIV		HHP		p-value
HIV cure achievable in next 10 years	55%	226/410	19%	26/140	< 0.001
HIV cure not achievable in lifetime	14%	56/410	16%	23/140	0.4
Aware of ATI	46%	182/399	62%	86/138	< 0.001
Ever participated/enrolled a patient in HIV cure-focused trial	5%	21/412	18%	25/140	< 0.001

*ATI* analytical treatment interruption, *HHP* HIV healthcare providers, *PLHIV* people living with HIV

## Discussion

We collected online responses from both PLHIV and HHP on their attitudes and perceptions towards ATI in HIV cure-focused research. While several studies have explored PLHIV motivations to enrol in clinical studies related to HIV cure [3, 4, 11, 12], quantitative analyses assessing HIV clinician attitudes towards ATI in the context of cure-related research have not been performed. Furthermore, we surveyed HIV clinicians, including primary care physicians who prescribe ART in the community, who were not necessarily involved in such research, to gain a broader sense of general perceptions on ATI from providers.

ATI is a critical component of HIV cure-focused clinical trials. PLHIV form strong therapeutic relationships with their providers so decisions about trial participation, particularly those that involve an alteration in their routine ART, will always need to involve their HHP. It is likely that PLHIV will follow the recommendation of their clinician about whether to enrol in such trials. Therefore, it is critical to understand the perspective of HHP providers at the same time as PLHIV attitudes towards ATI trials. We found a clear difference in awareness of ATI and optimism for an HIV cure between PLHIV and HHP.

A high level of participation in HIV research was noted amongst surveyed PLHIV, and a small number had previously enrolled in an HIV cure-focused study. In spite of scarce firsthand experience with HIV cure-related research; there was optimism among PLHIV that a cure would be achieved within the next 10 years. Importantly, this reflects current perceptions of HIV cure research among PLHIV, where previous qualitative studies have found mixed attitudes ranging from optimism to fear and disengagement [12, 13]. The observed lower optimism for HIV among clinicians may also expound reluctance to enrol patients in their care into a HIV cure-focused study. This may be explained by the adherence to universal recommendations for continuous ART, after The Strategies for Management of Antiretroviral Therapy (SMART) study (and other CD4 directed ART trials of the time) demonstrated an increased risk of serious opportunistic infections and all-cause mortality [1, 14]. The level of optimism in PLHIV and its disparity from HHP will be an important response to assess and compare over time and compare with that of HHPs, in particular as media reporting of HIV cure science over time may have given the impression that a cure is imminently achievable [8].

Motivations for PLHIV to enrol in HIV cure research involving ATI were mostly altruistic, with an emphasis on helping others above themselves. This has been demonstrated in multiple other surveys of PLHIV who have already participated in cure research [11], and those who are yet to enrol [3–5, 12, 15]. This is not so clearly apparent in other medical research fields, including paediatrics, where motivations include accessing unaffordable treatment and “enhanced care” [16–18] above altruistic reasons. This may be particularly evident in countries without a universal healthcare model. This was also noted in participants in cancer studies where only between 25 and 47% of surveyed participants stated that they enrolled for altruistic reasons [19, 20]. PLHIV have historically worked closely with scientists and researchers, advocating heavily for investing in research, particularly in the early ART studies [21]. This altruism of participating in research can also be seen as a form of activism.

The surveys were accessed online and only available in English. This limited access to potential respondents who reside in low- or middle-income countries (LMIC) where internet access is not universal and English is not the first language. As such, there was a bias in both the PLHIV and HHP surveys to respondents from Western and high-income countries, where much of the current HIV cure-related research is being conducted [1]. It is important for both clinical and social studies to be performed in LMIC where the greatest burden of infection is centred, and where many PLHIV have limited access to ART [22].

As the surveys were hosted on a website dedicated to HIV cure, there may be a bias towards PLHIV and HHP who are already interested or involved in HIV cure research. Furthermore, the surveys did not give participants the opportunity to expand on answers in free text comments. Thus the true motivations of PLHIV to enrol in such studies or detailed concerns from physicians around trial participation may not have been elucidated. While qualitative surveys have the benefit of exploring these issues in more detail, our quantitative study allowed for a large sample of participants to be surveyed. We have identified important themes for further investigation such as potential caution practiced by providers around HIV cure studies.

Both surveyed PLHIV and HHP were interested in HIV cure science. This positive perception is encouraging for investigators planning such studies and highlights the importance of engaging PLHIV and HHP to improve trial understanding and aid enrolment. Further research should be conducted in these populations to monitor how these attitudes may shift over time. Finally, as an important point of access for information for PLHIV, it is essential that up-to-date and accurate data from cure-focused studies are disseminated to all prescribers of HIV therapy, not only to clinicians who are already very interested in this research. Developing and broadening this interest in clinicians may assist in clarifying concerns and anxieties related to ATI, which will have a flow on effect to PLHIV as potential trial participants.

## Conclusion

Surveyed PLHIV and HHP demonstrated great interest in HIV cure-related research, however few respondents had previous experience participating in, or enrolling someone in an ATI study. HHP were less optimistic of the prospects of achieving an HIV cure compared to PLHIV, and this may curb enthusiasm to enrol their patients in such a study. This highlights HIV clinicians as an important target along with PLHIV for specific educational strategies aimed at increasing awareness and understanding of HIV cure-focused research.

## Data Availability

The survey questions are available as supplementary data. The anonymous datasets used and/or analysed during the current study are available from the corresponding author on reasonable request.

## Abbreviations

ATI: Analytical treatment interruptions
ART: Antiretroviral therapy
PLHIV: People living with HIV
HHP: HIV healthcare providers
LMIC: Low- or middle-income countries

## References

[1] Lau JSY, Smith MZ, Lewin SR, McMahon JH (2019). Clinical trials of antiretroviral treatment interruption in HIV-infected individuals. AIDS.

[2] Julg B, Dee L, Ananworanich J, Barouch DH, Bar K, Caskey M (2019). Recommendations for analytical antiretroviral treatment interruptions in HIV research trials-report of a consensus meeting. Lancet HIV.

[3] Dube K, Evans D, Sylla L, Taylor J, Weiner BJ, Skinner A (2017). Willingness to participate and take risks in HIV cure research: survey results from 400 people living with HIV in the US. J Virus Erad..

[4] Simmons R, Kall M, Collins S, Cairns G, Taylor S, Nelson M (2017). A global survey of HIV-positive people's attitudes towards cure research. HIV Med.

[5] Arnold MP, Evans D, Vergel N (2015). Recruitment and ethical considerations in HIV cure trials requiring treatment interruption. J Virus Erad.

[6] Dubé K, Evans D, Dee L, Sylla L, Taylor J, Skinner A, Weiner BJ, Greene SB, Rennie S, Tucker JD (2018). We need to deploy them very thoughtfully and carefully: perceptions of analytical treatment interruptions in HIV cure research in the United States—A qualitative inquiry. AIDS Res Hum Retroviruses..

[7] Protiere C, Spire B, Mora M, Poizot-Martin I, Preau M, Doumergue M (2017). Patterns of patient and healthcare provider viewpoints regarding participation in HIV cure-related clinical trials. Findings from a multicentre French survey using Q methodology (ANRS-APSEC). PLoS ONE.

[8] Power J, Fileborn B, Dowsett GW, Lucke J, Brown G, Ellard J (2017). HIV cure research: print and online media reporting in Australia. J Virus Erad.

[9] Lau JSY, Smith MZ, Allan B, Martinez C, Power J, Lewin SR, McMahon JH (2020). Perspectives on analytical treatment interruptions in people living with HIV and their health care providers in the landscape of HIV cure-focused studies. AIDS Res Hum Retroviruses..

[10] HIV Cure. Website for the Australian HIV Cure Community Partnership 2019. Website for the Australian HIV Cure Community Partnership. 2019. https://hivcure.com.au/. Accessed 08 April 2019.

[11] McMahon JH, Elliott JH, Roney J, Hagenauer M, Lewin SR (2015). Experiences and expectations of participants completing an HIV cure focused clinical trial. AIDS.

[12] Power J, Westle A, Dowsett GW, Lucke J, Tucker JD, Sugarman J (2018). Perceptions of HIV cure research among people living with HIV in Australia. PLoS ONE.

[13] Sylla L, Evans D, Taylor J, Gilbertson A, Palm D, Auerbach JD (2018). If We Build It, Will They Come? Perceptions of HIV cure-related research by people living with HIV in four U.S. Cities: a qualitative focus group study. AIDS Res Hum Retroviruses..

[14] El-Sadr WM, Lundgren J, Neaton JD, Gordin F, Abrams D, Strategies for Management of Antiretroviral Therapy Study G (2006). CD4+ count-guided interruption of antiretroviral treatment. N Engl J Med..

[15] Henderson GE, Peay HL, Kroon E, Cadigan RJ, Meagher K, Jupimai T (2018). Ethics of treatment interruption trials in HIV cure research: addressing the conundrum of risk/benefit assessment. J Med Ethics.

[16] Fisher JA (2007). "Ready-to-recruit" or "ready-to-consent" populations?: informed consent and the limits of subject autonomy. QualInq.

[17] Tromp K, Zwaan CM, van de Vathorst S (2016). Motivations of children and their parents to participate in drug research: a systematic review. Eur J Pediatr.

[18] Fisher HR. ‘Held together with human glue’: understanding participation in non-therapeutic paediatric randomised controlled trials. Doctoral dissertation. London: King’s College London, United Kingdom; Awarded 20 Dec 2012.

[19] Truong TH, Weeks JC, Cook EF, Joffe S (2011). Altruism among participants in cancer clinical trials. Clin Trials.

[20] Moorcraft SY, Marriott C, Peckitt C, Cunningham D, Chau I, Starling N (2016). Patients' willingness to participate in clinical trials and their views on aspects of cancer research: results of a prospective patient survey. Trials.

[21] Epstein S (1996). Impure science: AIDS, activism, and the politics of knowledge.

[22] UNAIDS. UNAIDS World AIDS Day Fact Sheet 2019. 2020. https://www.unaids.org/sites/default/files/media_asset/UNAIDS_FactSheet_en.pdf. Accessed 22 Jan 2020.

